# Invasive pulmonary fungal infections in patients with connective tissue disease: a retrospective study from northern China

**DOI:** 10.1590/1414-431X20165531

**Published:** 2016-09-22

**Authors:** H.F. Ge, X.Q. Liu, Y.Q. Zhu, H.Q. Chen, G.Z. Chen

**Affiliations:** 1 Department of Dermatology, The Affiliated Hospital of Qingdao University, Qingdao University, Qingdao, Shandong, China; 2 Laboratory Department, The Affiliated Hospital of Qingdao University, Qingdao University, Qingdao, Shandong, China

**Keywords:** Connective tissue diseases, Invasive pulmonary fungal infection, Pulmonary disease, Retrospective study

## Abstract

Invasive pulmonary fungal infection (IPFI) is a potentially fatal complication in patients with connective tissue disease (CTD). The current study aimed to uncover the clinical characteristics and risk factors of patients with IPFI-CTD. The files of 2186 CTD patients admitted to a single center in northern China between January 2011 and December 2013 were retrospectively reviewed. A total of 47 CTD patients with IPFI were enrolled into this study and assigned to the CTD-IPFI group, while 47 uninfected CTD patients were assigned to the control group. Clinical manifestations were recorded, and risk factors of IPFI were calculated by stepwise logistical regression analysis. Forty-seven (2.15%) CTD patients developed IPFI. Systemic lupus erythematosus patients were responsible for the highest proportion (36.17%) of cases with IPFI. *Candida albicans* (72.3%) accounted for the most common fungal species. CTD-IPFI patients had significantly elevated white blood cell count, erythrocyte sedimentation rate, C-reactive protein and fasting glucose values compared to controls (P<0.05). Cough, sputum and blood in phlegm were the most common symptoms. Risk factors of IPFI in CTD included maximum prednisone dose ≥30 mg/day within 3 months prior to infection, anti-microbial drug therapy, and interstitial pneumonia. CTD patients who have underlying interstitial pneumonia, prior prednisone or multiple antibiotics, were more likely to develop IPFI.

## Introduction

Connective tissue disease (CTD) is a group of systemic autoimmune diseases that include systemic lupus erythematosus (SLE), rheumatoid arthritis (RA), polymyositis/dermatomyositis (PM/DM), systemic sclerosis (SSC), mixed connective tissue disease (MCTD), primary Sjögren's syndrome (pSS), and several others. Immunosuppressant drugs are widely used to treat CTD. However, the use of immunosuppressive agents combined with the disease’s immune-mediated pathogenesis makes CTD patients vulnerable to opportunistic infections (1–3). The incidence of opportunistic infections in CTD patients has been continuously increasing. Previous data reveals that the occurrence of SLE, RA, and PM/DM combined with an opportunistic infection was 40-57%, 17 and 11.5%, respectively (4–6).

Invasive fungal infection (IFI) is a common opportunistic infection, and is a critical, life-threatening complication in CTD patients. A previous study demonstrated that the mortality rate of PM/DM patients complicated by an infection was 27.7%, among which, more than 50% did not survive as a consequence of the underlying fungal infection (6). The lung is the most frequently involved organ in IFI. Deterioration caused by invasive pulmonary fungal infections (IPFI) is usually the direct cause of death in CTD (7). However, relatively few cases of IPFI have been described in CTD, and the risk factors remain incompletely explored.

In the present study, we retrospectively evaluated clinical characteristics including CTD classification, symptoms, fungal species, laboratory indicators of inflammation, the application of prednisone, and other immunosuppressive drugs in CTD patients presenting with IPFI at a tertiary hospital in northern China from 2011 to 2013. We aimed to uncover the major clinical manifestations and risk factors for IPFI in CTD.

## Material and Methods

### Ethics statement

The study was approved by the Ethics Committee of the Affiliated Hospital of Qingdao University. The requirements of informed consent were waived by the Institutional Review Board.

### Patients and study design

The files of 2186 CTD patients who were hospitalized in the Department of Rheumatology, Affiliated Hospital of Qingdao University, from January 2011 to December 2013 were reviewed. All patients fulfilled the current acknowledged criteria for CTD diagnosis. SLE was diagnosed based on the American College of Rheumatology (ACR) criteria (8). RA was diagnosed according to the ACR classification criteria (9). The diagnosis of MCTD was conducted using the classification criteria proposed by Sharp et al. (10). The diagnosis of PM/DM was established according to the classification criteria proposed by Bohan (11). pSS was determined by the international classification criteria of the European and American collaborative group in 2002 (12). In addition, the diagnosis of interstitial pneumonia was based on the criteria of the American Thoracic Society and the European Respiratory Society in 2002 (13).

IFI was diagnosed when at least one of the following three conditions were presented: 1) positive culture from normally sterile sites (e.g., blood or cerebrospinal fluid specimens) or deep tissue specimens, and/or the presence of budding yeast, hyphae, or pseudo-hyphae; 2) histopathological examination of samples collected by needle aspiration or from a biopsy specimen showing hyphae or yeast with evidence of associated tissue damage (i.e., either microscopically or unequivocally demonstrated by imaging analysis); 3) fungal colonization that was not classified as an invasive fungal infection unless this was supported by the same positive culture outcome from two independent occasions, or from clinical and/or radiological evidence and/or in response to anti-fungal agents (14). A total of 47 CTD patients were diagnosed with pulmonary fungal infection. These patients were assigned to the CTD-IPFI group, while 47 CTD patients without any evidence of infection were assigned to the control group. Patients from the control group were derived from the in-patient database, and were selected using a random number table. All cases in the present study were seronegative for human immunodeficiency virus.

### Laboratory indicators

All blood samples were drawn at the onset of fungal infection. Indicators including white blood cell (WBC) count, erythrocyte sedimentation rate (ESR), C-reactive protein (CRP), and fasting glucose were detected by laboratory technicians at the same time.

### Data processing

Clinical data including age, gender, disease duration, frequency and length of hospital stay, clinical manifestations, associated medical conditions, causative fungal species, imaging conditions, and medication profiles (e.g., antibiotics, corticosteroids and immunosuppressive agents) prior to the diagnosis of IPFI were recorded and compiled in spread-sheet format (Microsoft Office Excel 2003; Microsoft Corp., USA) for subsequent analysis.

### Statistical analysis

Numerical data are reported as the mean and range, which was dependent on the data type. Descriptive data were compared using Dunnett's *t*-test. Categorical variables were compared by the chi-square test or Fisher's exact test. Stepwise logistic regression models were used to calculate the odds ratios (OR) and 95% confidence intervals (CI). Statistical analyses were performed using the SPSS v.11.0 software program for Windows (USA). P<0.05 was considered to be statistically significant.

## Results

### General and clinical characteristics

A total of 47 IPFI patients with CTD were enrolled and assigned to the CTD-IPFI group, while an equivalent number of CTD patients without infection were selected and assigned to the control group. IPFI accounted for 2.15% of all CTD cases reviewed (n=2186). There were no significant differences in age, gender, disease duration, and the number of patients who took immunosuppressants (prednisone, cyclosporine, azathioprine, tacrolimus, etc.) between the two groups. However, the number of patients diagnosed with interstitial pneumonia or who were under multiple antibiotics differed significantly between both groups (P<0.05, Table 1).

**Table:**
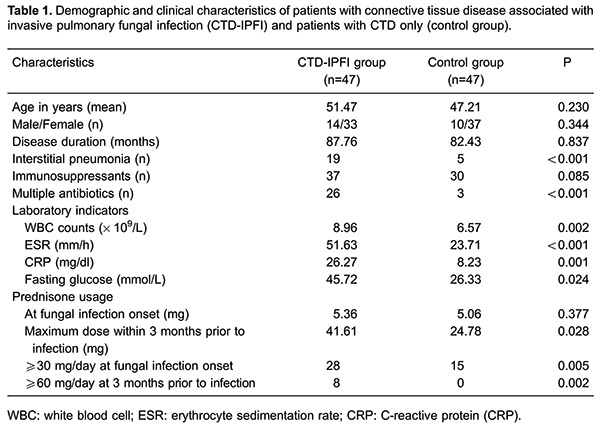

WBC counts, ESR, CRP and fasting glucose laboratory indicator levels were significantly greater in the CTD-IPFI group than in the control group (Table 1).

Patients in the CTD-IPFI group manifested a higher maximum dose of prednisone in the 3 months prior to fungal infection, compared with the control group (41.61 *vs* 24.78 mg, P=0.028); although the difference in prednisone dose at the onset of fungal infection between the two groups was not statistically significant (5.36 *vs* 5.06 mg, P=0.377). However, the number of subjects who received prednisone at ≥30 mg/day when fungal infection was diagnosed were significantly greater in the CTD-IPFI group, compared with the control group (28 *vs* 15, P=0.005). A similar pattern was observed in the number of patients who received prednisone ≥60 mg/day in the 3 months prior to infection (8 *vs* 0, P=0.002; Table 1).

### CTD types

In all patients diagnosed with IPFI, SLE outnumbered all the other types of CTD. The number and corresponding percentages of CTD-IPFI patients diagnosed with different CTD types are reported in Table 2.

**Table:**
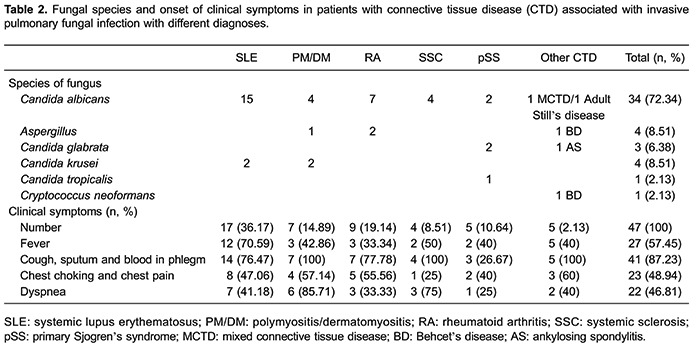

### Fungal composition

The most frequent causative pathogen was *Candida albicans* (34/47), which accounted for 72.34% of all cases with IPFI. This was followed by *Aspergillus* and *Candida krusei* infections (both 4/47). *Candida glabrata*, *Candida tropicalis* and *Cryptococcus neoformans* occurred less frequently than the above pathogen in CTD patients (Table 2).

### Clinical symptoms

Respiratory symptoms including cough, sticky sputum and blood-stained sputum were the most common symptoms in CTD patients with IPFI. Specifically, 41 of 47 patients presented such respiratory tract symptoms, which accounted for 87.2% of all CTD-IPFI cases. Symptoms, such as fever, chest thrusts and pain, and dyspnea, presented in approximately half of the CTD-IPFI patients (Table 2).

In severe cases, hemoptysis, dyspnea and hypoxemia were also observed. Rales could be heard on lung auscultation. In addition, chest radiographs or computed tomography scans showed diffuse patchy airspace shadowing of the lungs, multiple pulmonary nodular shadows, diffuse miliary nodules, or ground-glass-like changes.

### Risk factors of IPFI

All variables in Table 1, except laboratory indicators, were included in a backward-stepwise logistical regression model to identify risk factors for the presence of IPFI in CTD patients. The analysis revealed that a maximum prednisone dose ≥30 mg/day during the fungal infection, multiple antibiotics, and concurrent interstitial pneumonia were confirmed to be risk factors for IPFI in CTD patients (Table 3).

**Table:**
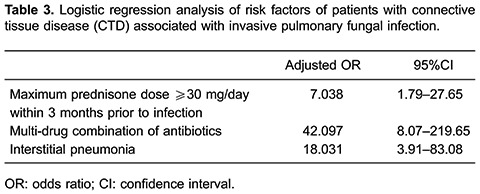

## Discussion

The present study evaluated the clinical characteristics and risk factors of IPFI in CTD patients. The results demonstrate that SLE accounted for most of the affected cases, and *C. albicans* was the most common pathogen. In addition, underlying interstitial pneumonia and the use of immunosuppressive drugs were risk factors for developing IPFI in CTD patients.

CTD is the third most common underlying disease of IFI following lung and neurological diseases (15). Among all types of CTD, SLE patients were more likely to develop IFI. A previous study demonstrated that infection rates were significantly higher in SLE patients than in RA patients (16). This result was consistent with the findings in our study. We found that SLE alone accounted for 36.17% of all fungal infected cases in CTD. Furthermore, it was also found that SLE, combined with PM/PA and RA, accounted for nearly 2/3 (68%) of all IPFI patients in the CTD group. There might be several explanations for this phenomenon. Due to the higher incidence of SLE in China, the number of IPFI cases in SLE patients may remain high, compared with other CTD diseases. In addition, the widespread use of corticosteroid therapy and/or immunosuppressive agents in SLE might be another reason for the high incidence of SLE in fungal infected CTD patients.

In the present study, *C. albicans* was responsible for 72.3% of all fungal infectious agents. This percentage was followed by *C. krusei* and *C. glabrata*, and the incidence of both *C. tropicalis* and *C. parapsilosis* were rare. These results were in line with the previous reports conducted by Pillay et al. and Sieving et al. (17,18). Moreover, studies in the United States have also reported that *Candida* species were dominant pathogens (19). However, in Taiwan and Korea, the most prevalent pathogens were *Cryptococcus* and *Aspergillus*, respectively (20,21). In the present study, we found that infection with *Candida* species other than *C. albicans* accounted for 17% of all infected cases. This observation was consistent with work of Bassetti et al. and Warnock (22,23). The increase in candidemia caused by non-albicans species was correlated with the increased use of azoles for prophylaxis or empirical treatment (23).

To date, predisposition factors for IPFI in CTD patients have not been fully determined. The present study suggests that prednisone doses exceeding 30 mg/day at the time of the infection, anti-microbial drugs use and an underlying interstitial pneumonia are three important risk factors of CTD-associated IPFI. This result implies that physicians should be alert to these possible risk factors in clinical practice. Immunosuppressive agents not necessarily increase the probability of a fungal infection, but they suppress the immune response (24). Recent studies on SLE also pointed out that the accumulated dose of glucocorticoids was associated with fungal infection (25,26). High-doses of glucocorticoids are marked risk factors for CTD-associated IPFI, and it involves several aspects of the mechanism of inflammation and infection. First, it suppresses cell-mediated immunity in humans (27,28). Second, corticosteroids inhibit the recruitment of neutrophils and monocyte-macrophages to the inflammatory sites, and depress the bactericidal functional activity of monocytes and neutrophils (29–31). Third, corticosteroid therapy could also change the structure and function of lymphocytes, which could suppress protective antibody synthesis and inhibit bioactive interferon production, thus, predisposing patients to opportunistic infections (32). It is therefore recommended that immunosuppressive therapy should be ceased under conditions of an active fungal infection.

Broad-spectrum antibiotics are usually administered to control a wide range of infection, or when a specific pathogen has not been determined. However, these might promote the propagation of drug-resistant bacteria and alter microbial flora constitution, resulting in fungal propagation (33). Our results revealed that 87.2% of patients in the CTD-IPFI group were administered broad-spectrum antibiotics for more than 7 days. The average duration of antibiotic therapy was 15.1 days, and 28 patients were given two or more antibiotics. The relationship between antibiotics and IFI has been verified in several other studies on patients with SLE. However, unlike other publications, Vinicki et al. (34) claimed that previous immunosuppression with azathioprine was the only risk factor associated with the development of IFI in Argentine patients with SLE. The heterogeneity in the study populations may explain the diversity of results.

Patients with underlying lung diseases such as interstitial pneumonia tend to have secondary infections, which precipitate respiratory failure and even death. In this study, interstitial pneumonia was one of three risk factors for IPFI in CTD. It is noteworthy that the widespread use of glucocorticoid therapy in the treatment of interstitial pneumonia also promote secondary fungal infections (35).

IFI is challenging to diagnose due to its insidious onset, rapid progression and the long time required for obtaining accurate etiological information. Without timely therapeutic interventions, the mortality rate of IPFI has reached 30-80% (36). Therefore, early and accurate diagnosis plays an important role in the management of IPFI. Traditional laboratory methods used for detecting funguses rely on a time-consuming morphological process. Currently, non-culture methods and molecular biology methods have greatly assisted the clinical diagnosis of fungal diseases. For patients with risk factors for IPFI, an empirical anti-fungal therapy may provide a prognostic benefit. An evaluation form (i.e., the Multi-Disease Risk Assessment) was used to assess risk factors for deep fungal infection at the West Virginia University Hospital in the United States, which was recommended to be undertaken in advance to determine high-risk patients and consider the necessity of appropriate preventive therapy.

There are several limitations in the present study. First, this single-center study had a small sample size and the characteristics of the study population might differ from other studies. Second, some of the important clinical characteristics were not recorded due to the retrospective design of the study. Third, we did not evaluate the effect of anti-fungal therapy or prophylactic intervention in the present study.

In summary, the current study revealed that CTD patients who have underlying interstitial pneumonia and previously received prednisone or multiple antibiotics were more likely to develop IPFI.
